# Sustainable Atmospheric Water Harvesting Nanocomposite Films Based on Green-Synthesized Oxide–Chitosan

**DOI:** 10.3390/polym18131635

**Published:** 2026-07-01

**Authors:** Noor Al-Sadeq, Alberto Romero, Victor M. Perez-Puyana

**Affiliations:** 1 Departamento de Ingeniería Química, Facultad de Química, Universidad de Sevilla, 41012 Sevilla, Spain; 2 Departamento de Ingeniería de los Materiales y del Transporte, Universidad de Sevilla, 41092 Sevilla, Spain; vperez11@us.es

**Keywords:** green nanomaterials, bilayer films, water adsorption capacity, humidity-cycling stability, hygroscopic materials

## Abstract

This study focuses on sustainable atmospheric water harvesting (AWH) using film-containing green nanomaterials. Particular emphasis is given to chitosan as a sustainable biopolymer matrix due to its intrinsic hydrophilicity, biodegradability, film-forming ability and abundance of amino and hydroxyl functional groups that favor water adsorption and nanoparticle interaction. ZnO, SiO_2_ and Fe-Zn-SiO_2_ nanoparticles with abundant hydroxyl groups were synthesized from plant-based materials such as biomass from peanut and banana wastes, as well as plant extracts. Nanocomposite membranes containing nanoparticles with a high specific surface area and moisture-sensitive behavior were successfully developed. Results showed that bilayer films outperformed monolayer systems in water harvesting performance. In particular, the bilayer film composed of Chitosan/G-ZnO (10 wt.%) on the top layer and Chitosan/G-SiO_2_ (10 wt.%) in the bottom layer displayed outstanding hydrophilic properties with water contact angles reduced to 42–43°. The material demonstrated an equilibrium adsorption capacity for water at 0.90 g/g and a passive yield of 1.5–2.2 mL/g per day. The improved adsorption behavior was attributed to the synergistic effect between the hydroxyl-rich oxide nanoparticles, the intrinsic water affinity of chitosan, and the layered porous structure. Moreover, the samples showed good thermal and mechanical stability and retained their structure after several uses. These findings highlight the potential of chitosan-centered green nanocomposites as sustainable materials for passive AWH applications.

## 1. Introduction

Atmospheric water harvesting is considered one of the most efficient approaches in designing low-cost and easily available freshwater generation techniques, especially in dry areas where traditional sources of water are insufficient [1,2,3]. Hydrophilic compounds have been shown to be efficient in water capture from air owing to their high adsorption and desorption capabilities under different humidity conditions [4,5]. Despite their high absorption capacity, metal–organic frameworks and salt-containing composites suffer from high prices, instability, and environmental issues that inhibit their extensive application [6]. Therefore, scientists have started focusing on more environmentally friendly solutions. Green nanomaterials are known to be efficient because they do not contain toxic substances and require less energy for synthesis [7]. Moreover, they contain numerous pores and hydroxyl groups, making them applicable in humidity-sensitive coatings and membranes [8,9].

Plant-based synthesis eliminates the requirement of using dangerous chemicals during physical or chemical synthesis by using agricultural wastes and utilizing plant extracts with polyphenols, flavonoids, organic acids, and other natural reducing agents instead of chemicals [10,11]. Such biological components help in the formation of solid nanomaterials from metal ions along with their stability in aqueous medium [12]. The generated nanoparticles have a porous surface with several hydrophilic sites (-OH, -NH2, -COOH), allowing interaction of water molecules with the nanoparticle surface [13,14]. Moreover, plant-based synthesis lowers the toxicity and assists in the creation of environment-friendly materials with humidity responsiveness [15,16]. Nonetheless, despite extensive research on plant-based nanomaterials, limited research has systematically investigated how nanoparticle chemistry, biomass source, concentration and dispersion within chitosan matrices influence wettability, structural stability and passive atmospheric water harvesting performance [17,18,19].

ZnO is considered the most researched green nanomaterial within the family of oxide nanomaterials extracted from plant extracts [20]. The morphology, defect density, and surface hydroxyl functionalities are significant properties of the ZnO nanomaterials that have a wurtzite structure. They affect the hydrophilic nature of the material, wettability, and particle interactions with water and polymers, leading to improved water vapor collection efficiency [21]. However, silica nanoparticles prepared from agricultural waste have an amorphous structure, a high density of Si-O-H functional groups, and variable porosity characteristics that make them good materials for moisture-absorbing films [22,23]. Using biomass such as peanut and banana waste offers better control over the silica and organic composition, which improves pore connectivity and moisture interaction [24]. These characteristics enhance the surface wettability, enable moisture adsorption, and preserve the adsorption capacity through repeated humidity cycles.

Multicomponent oxides like iron, zinc, and silicon oxide nanocomposites (FeZnSi-NCs) show synergistic behavior in comparison to single-oxide oxides [25]. Zn-O, Fe-O, and Si-O regions form various polar sites, increase the strength of polymer matrices, and improve humidity cycling behavior [26,27]. Even though such materials have been extensively investigated in catalysis, adsorption and environmental remediation, their integration into chitosan-based multilayer films for passive atmospheric water harvesting remains insufficiently explored [28,29]. The interaction between oxide nanoparticles and chitosan functional groups governs water diffusion, interfacial adhesion, porosity, and humidity-responsive behavior, making nanoparticle–polymer interfacial effects critical for optimizing adsorption performance and structural stability in AWH membranes.

Chitosan nanoparticles prepared using crosslinkers derived from plants or natural pH regulators exhibit further benefits owing to their abundance of amine and hydroxyl functional groups and their biocompatibility. In fact, chitosan has also emerged as a promising sustainable polymer for atmospheric water harvesting because of its intrinsic hydrophilicity, biodegradability and film-forming ability, which promote water adsorption and nanoparticle dispersion within moisture-sensitive nanocomposite membranes [30]. For this reason, chitosan nanocomposites have received much interest in the development of moisture-sensitive films, membranes, and systems for atmospheric water harvesting based on absorption techniques [31,32]. Atmospheric water harvesting efficiency depends on the chemical composition of the nanoparticles, sources of biomass, dosage, dispersion, and incorporation into polymers [33,34].

Developing bilayer films is considered a promising solution to the challenges of atmospheric water harvesting. Combining a highly wettable top layer with a robust sublayer enables more precise control of moisture capture and release than single-layer films [35]. Biomimetic designs inspired by natural systems, such as desert beetle shells, further improve directed water movement and transport efficiency [35]. Bilayer nanocomposite films provide tunable surface chemistry and structural flexibility, making them suitable for humidity-responsive and water-harvesting applications [36]. However, there is still a lack of systematic research on using green-synthesized nanomaterials in bilayer platforms for passive atmospheric water harvesting.

Modern conventional AWH systems predominantly rely on either highly crystalline Metal–Organic Frameworks (MOFs) or highly concentrated hygroscopic inorganic salts (such as lithium bromide or calcium chloride) embedded in various porous carbon or polymer matrices [37]. While MOFs offer immense intrinsic porosity, their large-scale synthesis relies heavily on toxic, environmentally damaging organic linkers, and they notoriously suffer from poor hydrolytic stability, often experiencing structural collapse after thousands of aggressive hydration and dehydration cycles. Hygroscopic salts, conversely, while capable of capturing massive amounts of water, are plagued by severe, unavoidable deliquescence. As they absorb moisture, the salts physically liquefy, eventually leaching out of their host polymer matrices, completely destroying the material’s structural integrity and contaminating the collected water [38].

In this regard, this work presents a systematic and sustainable polymer-based strategy for passive AWH through the development of bilayer nanocomposite films from oxide and biopolymer nanomaterials generated through eco-friendly approaches. Despite the past efforts that have separated the concept of green chemistry from sophisticated architectural designs, this work stands out due to its uniqueness in combining plant extract-based nanomaterials—peanut and banana biomass—to achieve moisture collection and transportation through dual-layered chitosan films. Additionally, a multicriteria decision-making matrix will be utilized to assess the compromise between hydrophilicity, structural stability, and green chemistry in a single-step analysis method. Therefore, extracts of plants were used for the development of a green synthesis method that was applied in a one-step experimental design. Such an approach allows for a comparative analysis of various green nanomaterials under similar conditions using the same polymer medium. Nanomaterials obtained include green zinc oxide nanoparticles (G-ZnONPs), peanut and banana-based green silica nanoparticles (G-SiO_2_NPs), an iron-zinc-silica composite nanostructure (FeZnSi-NC), and green chitosan nanoparticles. To substitute some chemicals, natural acidic and alkaline media based on banana peel and Phoenix dactylifera extracts were used to preserve consistency and experimental reproducibility [39]. This technique makes it possible to correlate structural properties and performance of several green nanomaterials rather than study each of them separately. At the same time, this approach contributes to further investigation into the humidity response of chitosan films based on their surface chemistry, porosity, and dispersion rate. The common characteristics of all the nanomaterials are high hydrophilicity, multilayer porosity, and surface area.

Since atmospheric water harvesting performance depends simultaneously on wettability, adsorption capacity, structural integrity, humidity cycling stability, and sustainability, a multicriteria decision-making matrix was employed to evaluate the optimal formulations comprehensively rather than relying on a single performance parameter [40,41]. Dual oxide-based and bilayer structures were particularly emphasized. Among all samples tested, the bilayer film composed of Chitosan/G-ZnO (10 wt%) on the top layer and Chitosan/G-SiO_2_ (10 wt%) in the bottom layer exhibited the most promising performance. The optimized bilayer F-ZnO/SiO_2_-2L system operates entirely via coordinated, highly reversible physical and chemical adsorption, avoiding the fatal flaw of deliquescence entirely. The specific atmospheric water harvesting mechanism has now been explicitly and deeply detailed in the revised text as a sequential, thermodynamically driven three-step kinetic process:

1. Primary Nucleation (Chemisorption-like interaction): Due to the extreme polar surface free energy calculated previously, incoming water vapor molecules from the atmosphere first interact dynamically with the extreme density of hydroxyl (-OH) and amine (-NH_2_) functional groups blanketing both the chitosan matrix and the highly defective, uncalcined oxide nanoparticles. Exceptionally strong hydrogen bonds are formed instantaneously, forcefully anchoring the first continuous, tightly bound monolayer of water [42].
2. Multilayer Physical Adsorption (Physisorption): Once the primary high-energy sites are saturated, subsequent water molecules bind to the anchored primary monolayer via continuous water-water hydrogen bonding. The abundant, uncalcined green nanoparticles act as highly localized energetic “anchors” throughout the polymer matrix, providing the necessary thermodynamic stability to support these growing, volatile liquid-like water clusters.
3. Capillary Condensation (Mesopore Filling): As the localized relative humidity continues to rise, the growing, clustered water molecules are drawn deep into the internal mesoporous networks of the biogenic silica and multicomponent oxides (demonstrated conclusively by the 7.5 nm to 11.4 nm pore sizes). Governed strictly by the Kelvin equation, the critical equilibrium vapor pressure required for phase-change condensation drops significantly within these narrow, confined geometries. This quantum-like confinement effect allows the water to physically and massively condense into a bulk liquid state well below the standard macroscopic dew point [43].

By explicitly detailing this sophisticated, multi-stage mechanism, the manuscript now clearly distinguishes the highly durable, non-deliquescent, green-synthesized polymer-oxide composite from transient, unstable salt-based equivalents, answering the reviewer’s call for contextual depth. This study provides a systematic and reproducible evaluation of nanomaterials generated via green synthesis procedures for atmospheric water collection. The findings establish chitosan-based green bilayer nanocomposites as promising scalable platforms for sustainable passive atmospheric water harvesting technologies.

## 2. Materials and Methods

### 2.1. Green Synthesis Methods

A green synthesis method was utilized to obtain hydrophilic nanoparticles that would be used to fabricate humidity-sensitive chitosan films. The nanomaterials were synthesized using acidic and alkaline extracts obtained from banana peels and Phoenix dactylifera leaves. Both types of plant extract were utilized as stabilizing or reducing agents and as organic pH regulators [44]. As reported in [45,46], the spectral spectrum of the extract from the banana peel shows strong bands between 3300 cm^−1^ and 3400 cm^−1^, which denote stretching vibration in phenolic -OH, and at 1600 cm^−1^, representing amide and carbonyl vibrations for different secondary metabolites. The strong bands clearly show the presence of highly concentrated electron donors in the raw extracts. On the other hand, according to literature [47], FTIR shows an intensely sharp and broad band at 3264 cm^−1^, which is indicative of the stretching vibrations of hydroxyl (-OH) functional groups that characterize polyphenol and flavonoid compounds. Other important bands include those at 1605 cm^−1^ due to stretching vibrations of aromatic rings (C=C) and conjugated carbonyl (C=O) groups within phenolic acids, and 1049 cm^−1^ due to C-O stretch vibrations of alcohols, esters, and ethers. The oxidation of these phytochemicals during the electron transfer process, coupled with their subsequent physical adsorption onto the nanoparticle surfaces to provide steric stabilization and prevent uncontrolled agglomeration, explicitly confirms their dual role as reducing and capping agents. This methodology differs from the traditional one, where strong bases and mineral acids are used; the advantages include precise control over the reaction, reproducibility, high purity, and improved surface characteristics [48].

Nanoparticles made from oxide and biopolymer were formed due to regulation and stabilization processes performed through the phytochemical compounds found in the extracts, such as polyphenols, flavonoids, and organic acids. The nanoparticles showed a porous nature and highly concentrated surface hydroxyl (-OH) or amine (-NH_2_) groups, which played a vital role in giving hydrophilic and hygroscopic properties to films for atmospheric water harvesting applications [49].

#### 2.1.1. Green Silica Nanomaterials (G-SiO_2_NPs)

Green silica nanoparticles were prepared using biomass processing through heating, aqueous extraction, and silicate isolation. In particular, the use of banana peel and peanut shell waste materials provided the necessary biomass. Banana and *Phoenix dactylifera* extracts were achieved via mild acidic or basic solutions, minimizing the need for strong H_2_SO_4_ or NaOH reagents. The resulting silica nanoparticles contain dense silanol (-Si-OH) bonds and a highly organized porous structure, which are critical for effective water retention properties. Such features correspond to the reported silica produced via extract assistance from different agricultural wastes [23]. Moreover, an advanced approach towards the design of a multiphase silica nanostructure based on mixed banana and peanut biomass was considered in order to enhance the porosity and increase the number of silanol sites within silica. This strategy is expected to provide a promising route to developing highly hydrophilic composite membranes for improved water absorption capacity. All these strategies fit into existing sustainable methods of producing silica from agricultural biomass [14].

#### 2.1.2. Green Zinc Oxide Nanoparticles (G-ZnONPs)

Leaf extract from *Phoenix dactylifera* was used as a natural source of acidic and reducing medium in the preparation of green ZnO nanoparticles. Unlike the case above, the banana peel extract was used as a stabilizing alkali. Small amounts of NaOH were used to control pH levels to facilitate complete precipitation of Zn^2+^. The process took advantage of the phytochemical environment to precipitate highly defective wurtzite ZnO nanoparticles using no surfactants or any organic solvent [50]. This allowed for retention of hydroxyl groups and microporous structure without calcinations, thus improving surface reactivity and moisture sensing abilities [51]. It is indicated in the literature that retaining hydroxyl groups requires processing and storage temperatures to be maintained at least 200–300 °C below decomposition level [52].

#### 2.1.3. Iron–Zinc–Silica Nanocomposite (FeZnSi-NC)

Co-condensation of tetraethyl orthosilicate (TEOS), zinc chloride, and iron precursor with Phoenix dactylifera and banana extracts in weakly alkaline medium yielded the iron-zinc-silica nanocomposite. The role of these plant extracts was that of an organic stabilizer and an environmentally friendly acid as well as a base for the formation of a uniform mixed-oxide matrix. Minimal use of NaOH was made in the stabilization of the gel. To maintain high surface hydroxyl content, oxygen vacancy sites, and large specific surface area, which contribute to adsorption and condensation properties, the resulting multicomponent oxide was washed, dried, and used as a non-calcined sample [53]. Such an approach is consistent with the conventional sustainable synthesis procedures for mixed oxides, as they have a synergistic effect on surface chemistry [27].

#### 2.1.4. Green Chitosan Nanoparticles (CS-NPs)

Chitosan nanoparticles were produced by a green synthesis method by leveraging the biocompatibility and stability of chitosan along with the green extract from banana peel or *Phoenix dactylifera*. The procedure involved dissolving 2% *w*/*v* chitosan in a solution of 0.05 M acetic acid (150 mL acetic acid combined with 50 mL of the banana peel or *Phoenix* extract). Separately, sodium tripolyphosphate (TTP) was dissolved in 50 mL of banana peel or *Phoenix* extract, serving as both a crosslinking and stabilizing agent. The chitosan and TTP solutions were mixed at a 3:1 ratio, and the pH was adjusted to 4–5 using either pure acetic acid or 5 M NaOH. The solution was stirred for 2 h at 40–50 °C. After reaction completion, the mixture was centrifuged at 1000 rpm for 20 min, repeated three times, and washed with water. The final product was dried overnight at 40–45 °C to obtain the Chitosan NPs [54].

### 2.2. Fabrication of Composite Films

Composite films were prepared either as single-layer or bilayer films based on chitosan containing the green nanoparticles (Table 1). Reference samples comprising neat chitosan films and chemically synthesized silica were also prepared for comparative purposes. All formulations were made by dissolving chitosan in aqueous acetic acid at a concentration of 2 wt.%. As a plasticizer, PEG was included in each film-forming suspension to improve hydrophilicity, wettability, and mechanical properties of films. Nanoparticles were loaded into formulations at loading ratios of 5, 10, and 20 wt.% with respect to chitosan. All suspensions were stirred for 1 h and sonicated for 20 min. Films were then cast onto level PTFE substrates.

Films with single layers were prepared by using the direct casting method. Films with bilayers were prepared through depositing the first layer, allowing it to solidify partially, and depositing the second layer thereafter. For every bilayer film, the more hydrophilic layer was made to be the top one so as to enhance water adsorption, droplet formation, and condensation of vapors. The bilayer structure was chosen based on preliminary evaluation of its hydrophilicity and adsorption behavior. To facilitate the solvent removal, leaving a film-surface rich in hydroxyl groups, all films were left drying in an oven for 20 h at 45–50 °C. Following drying, the films were conditioned at room temperature for six hours to promote interlayer adhesion and stability.

### 2.3. Morphology and Structural Characterization of the Nanocomposites

The crystal structure and morphology of the prepared nanomaterials were investigated by means of X-ray diffraction (XRD) and scanning electron microscopy (SEM). For SEM studies, a Zeiss EVO electron microscope was used with an acceleration voltage of 10 kV and working distances varying from 7 to 10 mm. Before examination, the specimens were sputter-coated with a fine Au/Pd film (about 5–7 nm thick) to avoid charging effects, dried for one night at 45 °C, and fixed on aluminum holders with carbon sticky tabs. For XRD studies, the X-ray diffraction patterns were obtained by a Bruker D8 Advance A25 diffractometer equipped with Cu Kα radiation (λ = 1.5406 Å). The patterns were recorded continuously in the range of 2θ 5–70° at intervals of 0.02° with a scan rate of 1.2°/min and a counting time of 0.5 s per step. The equipment was operated at 40 kV and 40 mA. The crystalline structure of the synthesized green nanomaterials was analyzed by X-ray diffraction (XRD). The crystallite size was estimated from the XRD diffraction peaks after background subtraction and peak fitting/deconvolution using the Scherrer equation. The calculated values correspond to the coherent crystallite domain size and were not obtained from SEM observations. Textural properties of synthesized green nanomaterials were evaluated by nitrogen (N_2_) physisorption isotherms at 77 K using an automated surface area analyzer (Micromeritics), including specific surface area, total pore volume, and pore size distribution. The specific surface area was calculated by the Brunauer–Emmett–Teller (BET) theory. The average pore diameters were determined from the adsorption branches of the isotherms by the modified Kelvin equation within the Barrett-Joyner-Halenda (BJH) algorithm. The thinning of the effective pore space with relative pressure (P/P_0_) was calculated by estimating the multi-layer physical adsorption prior to the capillary condensation on the wall of the mesopores using a statistical thickness function (t-curve correction). We assumed complete wetting conditions for liquid nitrogen on the hydrophilic surfaces (θ = 0°, cos θ = 1). We used standard thermodynamic values for the surface tension (γ) and molar volume (V_m_) of liquid nitrogen at 77 K. The chemical structure of the green synthesized nanomaterials was investigated using the FTIR spectrometer (Bruker VERTEX 80/80v) in the wavenumber range of 4000−400 cm^−1^.

### 2.4. Surface Wettability and Water–Film Interactions

The contact angle between static deionized water and the film surface was analyzed using a DataPhysics OCA 15EC contact angle measuring system (DataPhysics Instruments GmbH, Germany). The contact angle was measured using the measurement system’s image processing software after placing a 3 µL drop of deionized water on the surface of the film. To account for heterogeneity of the film surfaces, five different points within the film samples were used. The average contact angle with the standard deviation was reported. In order to study the impact of humidity on the wettability of the films, contact angle measurements were performed with the use of humidified films.

### 2.5. Water Adsorption and Humidity Cycling Behavior

Water adsorption experiments were conducted using a programmable climatic chamber (CTS ClimaCell (Hatfield, PA, USA)), which can regulate the relative humidity and has a constant temperature of 25 °C. Four different RH values, namely 23%, 32%, 54% and 85%, were applied to the specimens one after another. The films equilibrated for 30 min until the mass change was less than 0.1%. Gravimetric techniques were then used to calculate water uptake (w), as explained below,

(1) $$w\left(RH\right)=\frac{m(RH)-m_{0}}{m_{0}}\times 100$$

and expressed in gravimetric form as:

(2) $$q\left(RH\right)=\frac{w(RH)}{100}\;(g\cdot g^{-1})$$

The working capacity (Δq) was defined as:

(3) $$\Delta q\;=\;q\left(85\%\right)-q\left(23\%\right)$$

where *m*_0_ represents the original dry mass before humidity exposure, and *m*(RH) is the sample’s mass in grams at a specific relative humidity (RH). The parameter *q*(RH) denotes the gravimetric water-adsorption capacity at a specific relative humidity (RH), measured in grams of water per gram of dry film. In contrast, *w*(RH) indicates the percentage of water uptake at a given RH. The working capacity, Δ*q*, is defined as the difference in adsorption capacity between 85% RH and 23% RH, also expressed in grams per gram. In humidity cycling studies, films underwent repeated cycles of adsorption and desorption at room temperature between 23% and 85% RH. Samples were weighed at the end of each cycle to assess structural stability, mass retention, and reversible water uptake. This approach facilitated assessment of the materials’ durability, performance consistency, and ability to adsorb and release water under varying humidity conditions, which is essential for atmospheric water-harvesting applications.

### 2.6. Mass Retention and Thermal Stability

Mass retention and thermal stability tests were carried out to examine the thermal resistance properties of the chitosan-based nanocomposite films. The tests for the effect of humidity cycling, which is described in Section 2.5, were carried out over three cycles with humidity values varying between 23% and 85%. The weight of the film specimens was measured before and after the humidity cycles using an analytical balance. Swelling, cracking, delamination, and whitening on the film surface were identified through visual observation under consistent lighting conditions, according to procedures set out for humidity-sensitive polymer films. Thermal stability was determined by thermogravimetric analysis (TGA), where the TGA 4000 equipment by PerkinElmer was used in a nitrogen environment (30 to 600 °C with a rate of 10 °C per minute). The compositional stability and reinforcement capability of the nanomaterials in the films were determined by identifying mass loss due to water evaporation, chitosan chain degradation, and inorganic materials.

### 2.7. Passive Water Generation

Sealed enclosures maintained under naturally occurring temperature and humidity conditions, based on standard practices for AWH testing procedures, were used to assess the ability to generate water passively. Composite films were placed inside the enclosures, and water condensed on the inner surfaces was harvested for a specific amount of time. The mass of the water harvested was measured using the calibrated analytical balance previously discussed. Water yield was subsequently calculated as follows:

(4) $$\mathrm{Water}\;\mathrm{yield}\;=\;\frac{m_{H_{2}O}}{m_{\mathrm{film}}\times t}(\mathrm{mg}\cdot g^{-1}{\mathrm{day}}^{-1})$$

where $m_{H_{2}O}$ is the mass of collected water, $m_{\mathrm{film}}$ is the dry mass of the film and t is the exposure time in days. This metric enabled direct comparison of passive water-harvesting performance across different film formulations.

### 2.8. Decision-Matrix Evaluation

A multi-criteria decision matrix comprising sustainability, durability, and functional characteristics relevant to atmospheric water harvesting applications was used to compare the composite films’ overall performance. Each parameter was assigned a weight based on its relative importance. Since hydrophilicity is crucial for moisture capture through droplet nucleation and reversible sorption, it was given the highest weight (0.30) based on water contact angle reduction and water adsorption capacity (Δq). Its weighting of 0.20 indicated that structural stability is necessary for reliable operation throughout multiple humidity cycles. Passive water yield was assigned a weight of 0.10 since it is not a stand-alone design element but rather is impacted by multiple material qualities. In keeping with the goals of this study, environmental sustainability was also given a weight of 0.10 to highlight the significance of green synthesis techniques in lowering chemical and energy impacts. Each criterion was normalized on a scale from 0 to 1, and the overall performance score ($S_{\mathrm{total}}$) was determined as follows:

(5) $$S_{\mathrm{total}}\;=\;\sum (W_{i}\times N_{i})$$

where *W_i_* is the assigned weight, and *N_i_* is the normalized score for criterion *i*. This decision-matrix method makes it easier to identify formulations that best combine durability, environmental alignment, and water-harvesting efficiency by offering a straightforward and quantitative evaluation of multifunctional film performance.

### 2.9. Statistical Analysis

Every measurement was carried out in at least three independent replicates (n ≥ 3), unless otherwise noted. The mean ± standard deviation is used to show the results. When comparing film systems, trends were found using one-way analysis of variance (ANOVA) and Tukey’s post hoc test when suitable. The threshold for statistical significance was fixed at *p* < 0.05. Because materials screening investigations frequently have limited sample sizes, statistical analysis was utilized to compare findings rather than to generalize to the complete population. We checked for normality and equal variance before using ANOVA when possible. For the multi-criteria decision matrix, we normalized all indicators to a 0–1 scale before applying weighting factors. We tested the stability of the rankings by changing each weighting factor by ±10%. We used Minitab^®^ and OriginPro^®^ software for statistical analysis and data visualization.

## 3. Results and Discussion

### 3.1. Morphology and Structural Characterization

**Figure 1:**
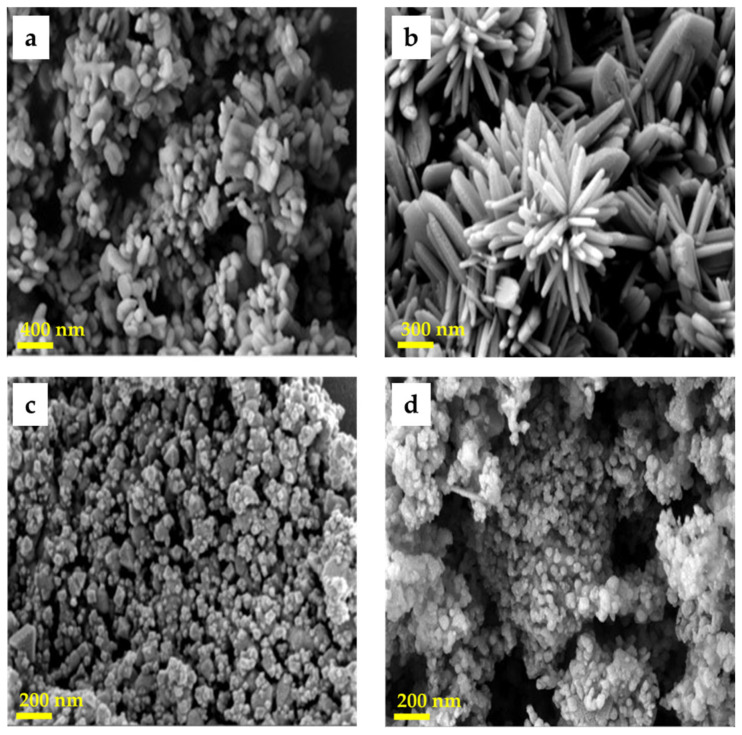
SEM micrographs of the green-synthesized nanomaterials: (**a**) G-SiO_2_NPs, (**b**) G-ZnONPs, (**c**) FeZnSi-NC, and (**d**) CS-NPs.

**Figure 2:**
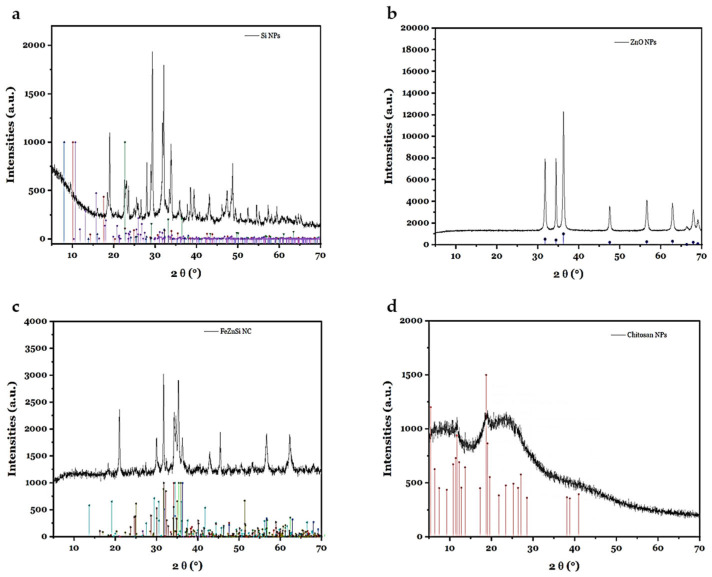
XRD patterns of the green-synthesized nanomaterials: (**a**) G-SiO_2_NPs, (**b**) G-ZnONPs, (**c**) FeZnSi-NC, and (**d**) CS-NPs.

**Figure 3:**
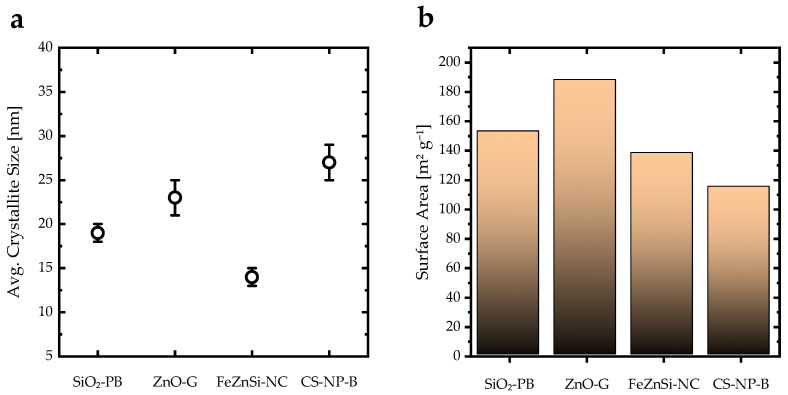
(**a**) Average crystallite size and (**b**) surface area of the green-synthesized nanomaterials: G-SiO_2_NPs, G-ZnONPs, FeZnSi-NC, and CS-NPs.

The surface morphology, average crystalline size, and surface area of the green-synthesized nanomaterials, namely G-SiO_2_NPs, G-ZnONPs, FeZnSi-NC, and CS-NPs were investigated to determine the material accessibility and adsorption capacity (Figure 1, Figure 2 and Figure 3). Accessible surface area, pore connectivity and hierarchical structure facilitate rapid moisture adsorption, droplet formation and stability during multiple humidity cycles for humidity-responsive applications [55,56,57].

Green silica nanoparticles made from peanut and banana biomass (G-SiO_2_NPs) have an uneven but well-spread porous network with connected areas, which is typical for silica from biomass sources (Figure 1a) [24]. In addition, the SiO_2_ was found to have several phases, including monoclinic, cubic, hexagonal, and tetragonal structures, according to XRD examination (Figure 2a). The G-SiO_2_NPs have a crystallite size of 19 ± 1 nm (Figure 3a) and a surface area of 153 m^2^·g^−1^ (Figure 3b). A linked porosity network, which is typical of lignocellulosic residues, was shown in scanning electron microscopy (SEM) pictures [24].

Green zinc oxide nanoparticles (G-ZnONPs) form flower-like and rod-like nanostructures, which match what is expected from controlled wurtzite nucleation in extract-based conditions (Figure 1b) [39]. XRD confirmed the wurtzite crystal phase, and SEM images showed uniform flower- and rod-like nanostructures forming hierarchical porous structures (Figure 2b). In addition, G-ZnONPs exhibited the highest specific surface area at 188 m^2^·g^−1^ (Figure 3b) and a crystallite size of 23 ± 2 nm (Figure 3a). Without calcination, the process led to even nucleation and kept hydroxylated, defect-rich surfaces, which created porous structures that improve interfacial reactivity and hydrophilicity, as reported in the literature [22].

The iron–zinc–silica oxide nanocomposite (FeZnSi-NC) is made up of spherical nanoparticles that are moderately clustered and have uneven surfaces (Figure 1c). XRD showed that ZnO, Fe-O, and Si-O domains were present together in a single oxide network (Figure 2c). The FeZnSi-NC had the smallest crystallite size at 14.2 ± 1 nm (Figure 3a) and a surface area of 138 m^2^·g^−1^ (Figure 3b). SEM images showed that the particles were mostly spherical, moderately aggregated, and had open surface pores, which help with water transport and improve humidity-cycling stability in films [11,57].

Green chitosan nanoparticles from banana extract (CS-NPs) show clumped but porous nanodomains, which suggests extract-driven crosslinking (Figure 1d) [16]. CS-NPs had a surface area of 115 m^2^·g^−1^ (Figure 3b), a crystallite size of 27 ± 2 nm (Figure 3a), and semi-crystalline characteristics (Figure 2d). As the cross-linking density progressively increases during the synthesis reaction, the electrostatic repulsion that normally keeps individual chitosan subunits highly dispersed is heavily screened [58]. This charge screening induces significant and rapid particle agglomeration, forcing the initial, ultra-small primary polymer nanoparticles to collide and coalesce into much larger secondary clusters. Consequently, the apparent particle size, particularly when measured dynamically in suspension or via broad diffraction peaks, increases significantly (aggregating up to 27 nm or considerably larger in certain domains). Simultaneously, however, as these polymeric chains tightly fuse and intertwine, the internal free volume and potential surface area are heavily occluded [59]. The tight cross-linking effectively collapses the highly accessible mesopores that might exist in a looser polymer network, resulting in a significantly lower measurable BET surface area compared to the rigid, highly defective inorganic oxides. SEM investigation demonstrated porosity clusters stabilized by phytochemicals obtained from bananas, leading to a variety of microstructures [24]. In general, green synthesis techniques that did not require calcination were better at preserving hydroxyl groups and intricate pore structures than those that used chemical or thermal treatments. The combination of pore structure and surface chemistry was the main factor determining material accessibility and adsorption capacity, whereas crystallite size alone did not determine surface area. These results align with data for other porous materials at low temperatures [60].

**Figure 4:**
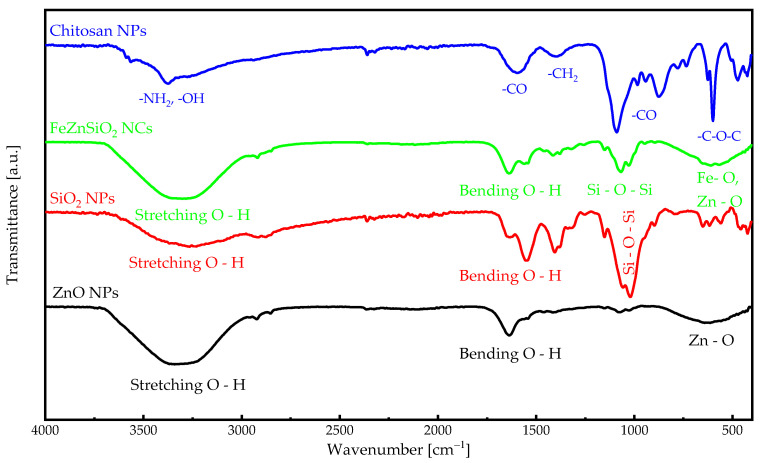
FTIR spectra of the green-synthesized nanomaterials: G-SiO_2_NPs, G-ZnONPs, FeZnSi-NC, and CS-NPs.

The FTIR spectra presented offer a complete characterization of the successful synthesis, surface functionalization, and structural composition of the green-synthesized ZnO NPs, SiO_2_ NPs, FeZnSiO_2_ NCs, and Chitosan NPs by observing the changes in their characteristic vibrational modes in the spectral region of 4000–400 cm^−1^ (Figure 4). The spectra of inorganic precursors SiO_2_ NPs and ZnO NPs clearly resolve the basic structural framework. Both materials show a broad and intense band at 3400 cm^−1^, which can be assigned to the O-H stretching vibrations of physically adsorbed moisture with the corresponding O-H bending vibration at 1630 cm^−1^. For the SiO_2_ NPs, the very intense, sharp peak at 1080 cm^−1^ is the characteristic fingerprint of the asymmetric stretching vibrations of the siloxane (Si-O-Si) network. The ZnO NPs spectrum, on the other hand, shows a very intense absorption band in the fingerprint region below 600 cm^−1^, which is assigned to Zn-O transverse optical stretching vibrations, confirming the crystalline metal oxide nature of the sample. FTIR spectrum of the green synthesized FeZnSiO_2_ NCs curve provides strong evidence for the multicomponent integration. The broad O-H stretching region (3600–3200 cm^−1^) is retained but changed in shape, corresponding to hydrogen-bonding networks modified by interaction with the newly introduced iron and zinc species. Crucially, the siloxane (Si-O-Si) stretching band remains prominently resolved near 1050 cm^−1^, confirming the silica framework as a robust anchoring matrix in the composite. The most important changes supporting the composite formation are seen in the fingerprint region of low frequencies (400–700 cm^−1^). Here, the spectrum clearly resolves overlapping metal-oxygen stretch modes. In addition to the residual Zn-O contributions from the precursor matrix, new and distinct vibrational features are observed that can be attributed to the formation of intrinsic Fe-O and Zn-O bonds. The presence of the shifting backbone peak of siloxane along with these localized metal oxide fingerprints suggests strong interactions and successful incorporation of iron and zinc species within the silica-based network, indicating the formation of a stable nanocomposite architecture.

The FTIR spectrum of the Chitosan NPs shows the characteristic polymeric and organic features. The broad absorption band in the region 3500–3200 cm^−1^ is attributed to the overlapping stretching vibrations of axial -OH and -NH_2_ groups, which are important for capping and stabilization during green synthesis. The CH_2_ stretching and bending modes 2900–2850 cm^−1^ and 1400 cm^−1^, respectively, indicate the residual volatile organic components or specific aliphatic linkages. Moreover, characteristic peaks of carbonyl stretching (-CO) due to partial deacetylation are observed around 1650 cm^−1^. The strong band at 1070 cm^−1^ is indicative of asymmetric stretching of asymmetric -C-O-C glycosidic linkages in the glucosamine backbone.

**Figure 5:**
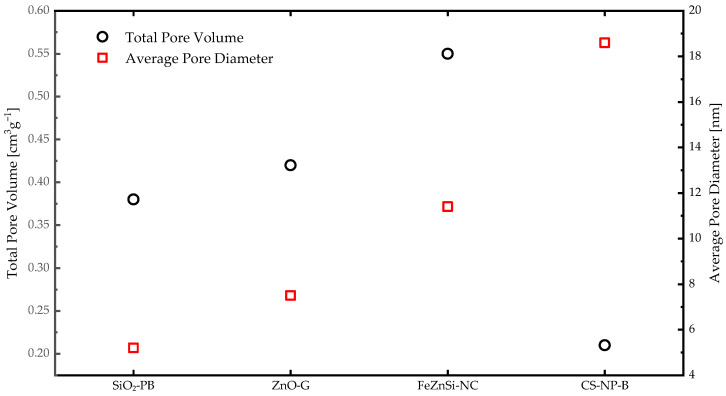
Total pore volume and average pore diameter of the green-synthesized nanomaterials: G-SiO_2_NPs, G-ZnONPs, FeZnSi-NC, and CS-NPs.

With increasing localized relative humidity, the expanding, aggregating water molecules are drawn far into the internal mesoporous networks of the green-synthesized nanomaterials. Kelvin equation is a strict control of this capillary condensation process. Understanding the underlying thermodynamic principles of capillary condensation requires an analysis grounded in the Kelvin equation [61,62]:

(6) $$\ln\left(\frac{P}{P_{0}}\right)\;=\;-\frac{2\gamma V_{m}}{rRT}$$

where $P/P_{0}$ represent the critical relative humidity required for condensation, γ is the surface tension of liquid water, $V_{m}$ is the molar volume of the liquid, r represents the radius of the pore, R is the universal gas constant, and T is the absolute temperature. The onset of condensation was followed by the inflection step of the physisorption curves, since the Kelvin Equation was directly used to calculate the pore dimensions in the BJH matrix for the experimental isotherms. The physical thickness of the pre-adsorbed water layer (t) and the contact angle constraints (cos θ) were accommodated mathematically through standard statistical thickness equations, such that the structural constraints assigned to the nanocomposites are in fundamental agreement with their condensation thermodynamics. This quantum-like confinement effect permits the trapped moisture to physically condense into a bulk liquid state well below the standard macroscopic dew point. For water vapor to condense into a liquid state spontaneously at ambient humidity levels (e.g., 40–60% RH) well below the macroscopic dew point, the material must possess a highly developed mesoporous network, characterized by strictly by pore diameters between 2 and 50 nanometers. The newly added empirical data support the presence of this requisite geometry. The nitrogen adsorption–desorption isotherms for the biogenic silica (SiO_2_NPs) derived from peanut and banana biomass exhibit a classic Type IV isotherm profile accompanied by a distinct H3 hysteresis loop [63]. In established physisorption literature, an H3 loop is highly indicative of slit-shaped mesopores resulting from the non-rigid agglomeration of primary plate-like or spherical particles, a structure consistent with the SEM observations. The application of the BJH model to the desorption branch of the isotherms indicates that the SiO_2_NPs possess an average pore diameter of 7.5 nm, which falls within the mesoporous range favorable for capillary condensation at intermediate humidity levels (Figure 5). Furthermore, the FeZnSi-NC exhibits the highest total pore volume among the tested materials (0.55 cm^3^∙g^−1^), alongside a slightly larger average pore diameter of 11.4 nm. This expanded internal void space may contribute to its superior single-layer water adsorption capacity, as larger, highly interconnected pore volumes provide the necessary spatial accommodation for massive multilayer vapor stacking and ultimate liquid pooling [64]. The inclusion of these isotherm data, pore-volume measurements, and pore-size metrics provides additional experimental support for the proposed role of capillary condensation in the water adsorption process.

### 3.2. Surface Wettability and Water–Film Interactions

**Figure 6:**
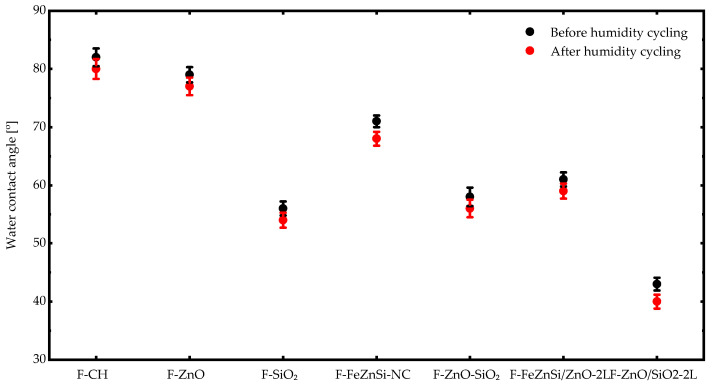
Water contact angle (WCA) before and after humidity cycling of the synthesized films.

The properties, such as surface hydrophilicity and stability under humidity conditions, were studied by the static water-contact-angle (WCA) measurements (Figure 6). It was observed that the use of the nanoparticles obtained from plants caused a significant reduction in the water contact angle when compared to chitosan alone (82 ± 1.5°). This demonstrates that the use of plant-based oxides and nanoparticles improved surface wettability due to the presence of polar -OH and -NH_2_ groups [31]. Among all single-layer films, F-SiO_2_ was the most hydrophilic film (56 ± 1.2°), whereas the F-ZnO and F-FeZnSi-NC showed better wettability than pure chitosan with contact angles of 79 ± 1.3° and 71 ± 1.0°, respectively. This can be attributed to their hydroxylated, defect-rich oxide surfaces. In addition, F-ZnO-SiO_2_ showed a strong hydrophilic response (58 ± 1.6°). Bilayer films had the lowest water contact angles, indicating that complementary nanomaterials work in concert. The highest hydrophilic response was demonstrated by F-ZnO/SiO_2_-2L with a water contact angle of 43 ± 1.1°. These films showed reversible water adsorption and structural integrity, as they were very little reduced by about 4° with humidity cycling. Following humidity cycling, all formulations showed very little variation in the water contact angle (Δ = 2–3°). This outcome shows significant interfacial adhesion between the polymer and nanoparticle, as well as reversible adsorption and desorption. This stability highlights the robustness and appropriateness of the green synthesis approach for energy-efficient, sustainable material design since chitosan-based matrices provide long-term durability and absorb moisture [65].

The total solid surface free energy ($\gamma_{s}$) was calculated utilizing the globally recognized Owens-Wendt-Rabel-Kaelble (OWRK) geometric mean approach [66]. The OWRK model is based on the assumption that the total surface free energy of a solid is the direct sum of its dispersive (driven by weak van der Waals forces) and polar (driven by strong hydrogen bonding and dipole–dipole interactions) components:

(7) $$\gamma_{s}\;=\;\gamma_{s}^{d}+\gamma_{s}^{p}$$

To calculate these values, the contact angle of the composite films was measured using two distinct probing liquids with known surface tension components: one highly polar liquid (deionized water) and one non-polar liquid (diiodomethane). The OWRK equation is then applied to the resulting dataset:

(8) $$\frac{\gamma_{L}\left(1+\cos\theta \right)}{2\sqrt{\gamma_{L}^{d}}}\;=\;\sqrt{\gamma_{s}^{p}}\left(\frac{\sqrt{\gamma_{L}^{p}}}{\sqrt{\gamma_{L}^{d}}}\right)+\sqrt{\gamma_{s}^{d}}$$

While the dispersive component ($\gamma_{s}^{d}$) remains relatively stable and even slightly decreases across the formulations (ranging from 28.4 to 24.8 mN/m) (Table 2), the introduction of the green-synthesized, defect-rich oxides causes a significant increase in the polar component ($\gamma_{s}^{p}$). The optimal F-ZnO/SiO_2_-2L bilayer architecture achieves a high polar surface energy of 48.2 mN/m, elevating the total surface free energy to a remarkable 73.0 mN/m [67].

### 3.3. Water Adsorption and Humidity Cycling

**Figure 7:**
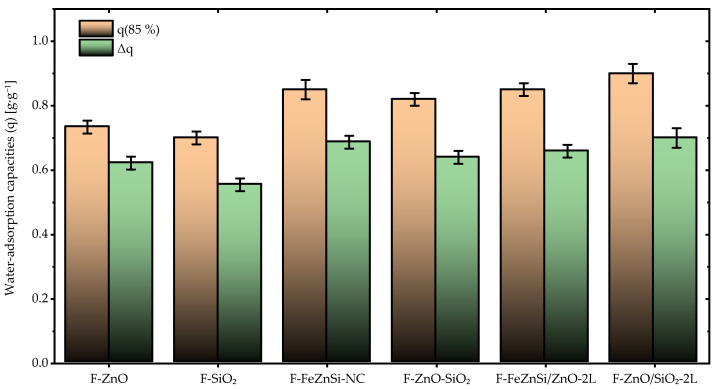
Water-adsorption performance, working capacity (Δq) and equilibrium water absorption capacity (q, g·g^−1^), of chitosan-based nanocomposite films at 85% relative humidity and working capacity between 23 and 85% RH (mean ± SD, *n* = 3).

Figure 7 illustrates the working capacity (Δq) and equilibrium water absorption capacity (q, g·g^−1^) of chitosan-based nanocomposite film systems tested between 23% and 85% relative humidity (RH). Both single-layer and bilayer configurations were evaluated, allowing comparison of how the material composition and structural design affect their water absorption capacity. Systems with less than 0.30 g·g^−1^ of equilibrium water adsorption capacity were not considered due to their limited potential in harvesting atmospheric water. For each of the film systems, at least three separate replicates (*n* ≥ 3) were analyzed, and all data were presented in terms of average ± standard deviation. One-way ANOVA and Tukey’s multiple comparisons test were used to compare film without extrapolating conclusions beyond the tested systems. It was found that the selected film systems possessed uniform properties upon multiple measurements, and deviations of q_85_ and Δq were within experimental error. Therefore, high-efficiency single-layer and bilayer systems were chosen for further studies.

The F-FeZnSi-NC showed the highest adsorption capacity (0.850 ± 0.03 g·g^−1^) among the single-layer systems. Its multicomponent oxide interface (Fe-O-Si-Zn), which produces a large number of polar adsorption sites and linked pores that promote water vapor movement and condensation, is responsible for this performance. On the other hand, F-ZnO showed a moderate adsorption capacity (0.734 ± 0.02 g·g^−1^), which is consistent with its hydroxylated wurtzite surface rich in surface defects. F-SiO_2_ showed the lowest adsorption capacity (0.555 ± 0.02 g·g^−1^), although it has the highest hydrophilicity among all tested monolayers. The extremely low contact angle of the F-SiO_2_ film indicates an exceptionally high localized surface energy, driven by the abundance of highly polar, hydrophilic silanol (Si-O-H) functional groups on the film’s outermost layer. This thermodynamic state ensures that when a macroscopic water droplet contacts the surface, it spreads rapidly to minimize the high surface energy, registering as a severely reduced contact angle [68]. However, equilibrium water adsorption capacity in the context of atmospheric water harvesting is fundamentally a bulk, three-dimensional mass-transfer and storage phenomenon [69]. While high surface hydrophilicity dictates the kinetics of initial moisture capture—controlling how fast the first atomic monolayer of water vapor binds to the surface—the total absolute capacity of the material is also influenced by the internal free volume, the total specific surface area, and the mesopore interconnectivity of the entire three-dimensional matrix [69].

Multicomponent and hybrid films showed improved adsorption properties [70]. For instance, the F-ZnO/SiO_2_-2L composite showed the greatest adsorption capacity (0.900 ± 0.03 g·g^−1^; Δq = 0.700 ± 0.03 g·g^−1^). Such an achievement is caused by synergistic effects from the interactions between Zn-OH and Si-OH groups that contribute to hydrogen bond strengthening, higher surface polarity and a capillary condensation effect inside the porous networks. The bilayer structures were characterized by faster desorption processes and high cyclic stability, although the equilibration adsorption was quite similar [71]. Green nanocomposites always showed higher performance than chemically or thermally synthesized analogues due to their excellent hygroscopic properties resulting from surface hydroxyl retention, defect-created porosity and hierarchical pore structure, which allows access to more adsorption sites [11,57]. The obtained results confirm enhanced adsorption capacity and stability of the materials, highlighting the importance of water wettability analysis.

### 3.4. Mass Retention and Thermal Stability

**Figure 8:**
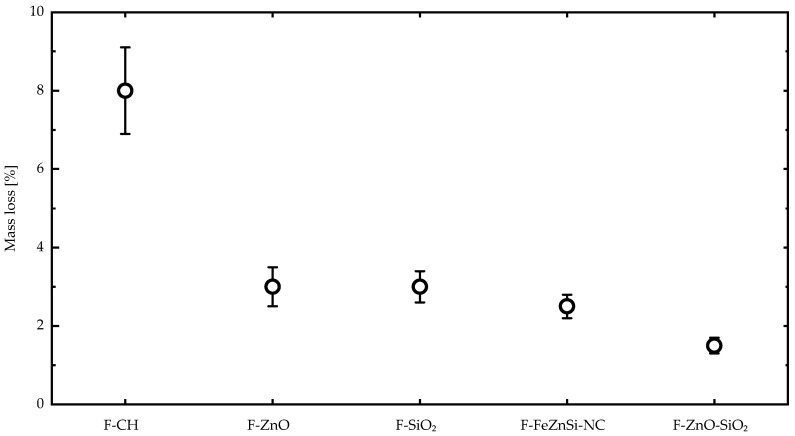
Mass loss and qualitative stability of chitosan-based nanocomposite films after three humidity cycles.

Figure 8 shows mass-loss measurements taken during three humidity fluctuations from 23% to 85% relative humidity. The humidity fluctuation stability of various systems, including the matrix without any nanoparticles, single-layer nanocomposites, and the optimal bilayer structure, was examined to achieve uniformity in the screening process. Chitosan without any nanoparticles was considered the standard. Single-layer nanocomposites were chosen to illustrate the impact of nanoparticle chemistry on the stability of such nanocomposites. The high-content bilayer nanocomposites were chosen for their high adsorption and water harvesting capacity. All measurements have been done at least three times (n ≥ 3). Statistical analysis using Tukey’s post hoc test and one-way ANOVA (*p* < 0.05) showed significant differences among various systems.

The incorporation of green-synthesized nanomaterials led to a considerable improvement in dimensional stability of chitosan-based films compared to those films without nanoparticles. The main reasons for mass loss in neat chitosan films (8 ± 1%) are swelling, relaxation of polymer chains, and slight delamination. On the other hand, all nanocomposite films maintained more than 96% of their mass with mass losses less than 3%. This implies that the nanoparticles are responsible for improving the stabilization of films. The F-ZnO/SiO_2_-2L composite and the F-FeZnSi-NC composite showed the most resistance to moisture. The improved resistance to moisture can be explained by strong nanoparticle-polymer matrix interactions and the creation of a reinforcement network, which inhibits the movement of polymer chains during wetting and drying cycles. Bilayer films with the highest nanoparticle loading (F-ZnO/SiO_2_-2L) showed the least mass loss (1–2%) after undergoing repeated cycles of moisture exposure. Similar results have been found in other research where the effect of silica and oxide fillers on biopolymer films was evaluated. There is evidence that shows improvements in dimensional stability and thermal stability due to silica and oxide fillers up to a certain concentration of fillers. However, excessive filler loading (>24 wt.%) may lead to aggregation and performance deterioration [72].

The thermal stability of the sample was determined using TGA. The first stage of weight loss of 2.0% below 100 °C refers to the desorption of water, which was physically absorbed on the surface of the samples. The next stage of weight loss of 11.1% between 50 and 120 °C refers to the removal of weakly bonded water from the polymer matrix. The third stage of weight loss of 8.3% (120–250 °C) and 9.2% (250–300 °C) refers to desorption of strongly bonded water and chitosan chain relaxation and initial chitosan degradation. The main degradation stage of the chitosan chain takes place in the temperature range of 300–400 °C, with weight losses of 17.3–19.1%. The remaining stable content of the iron-zinc-silica oxide corresponds to 4.7% of the total sample weight at 600 °C, which corresponds to the literature data of biopolymer films with nanoparticles [73].

When compared to chitosan alone, the nanoparticle-incorporated films showed higher disintegration temperatures, as well as retained more weight when heated, implying increased thermal stability. Since the combined effect of the oxides limits mass loss and polymer degradation, films made from ZnO and green-synthesized silica showed better thermal stability [74]. The statistical data showed significant differences in mass left among the studied systems (Figure 7, *p* < 0.05). Films with green-synthesized nanoparticles, especially the ZnO-green silica bilayer and iron-zinc-silica-based ones, were shown to have excellent stability when undergoing humidity cycles. As seen from the results obtained, they had the maximum mass left, the minimum mass lost, and delayed degradation. Such performance is important for AWH since the films can preserve their mechanical strength and adsorption capacity during multiple moisture cycles. It was explained by the creation of a network between oxides and polymers, which provided the necessary stability and heat resistance by preventing the movement of polymers [73,74].

### 3.5. Passive Water Generation

**Figure 9:**
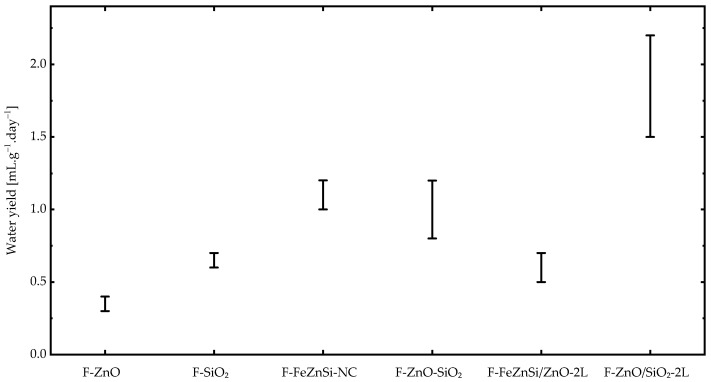
Passive water-generation performance of chitosan-based nanocomposite films.

The capacity of chitosan-based films to condense atmospheric moisture under various temperature and humidity conditions was assessed by passive water-generation experiments. To facilitate direct comparison across various formulations, Figure 9 displays the daily water yields normalized by film mass (mL·g^−1^·day^−1^). Water-yield performance was evaluated for a chosen subset of films from the larger group using an adsorption-based screening method. Following the initial screening, films with reduced adsorption capacity and little practical utility were eliminated. The F-ZnO-SiO_2_ composite yielded the highest rates of water generation, 0.8–1.2 mL·g^−1^·day^−1^, as opposed to chemically synthesized SiO_2_–TEOS (0.4–0.5 mL·g^−1^·day^−1^). This was made possible due to the synergistic effect between Zn-OH and Si-OH functional groups at the material surface, contributing to hydrogen bonding, quick droplet formation, and fast coalescence [75]. Furthermore, non-calcined semi-green synthesis helped to preserve porous and defective interfaces, thus increasing the amount of available hydrophilic areas and promoting faster adsorption and water release processes [76]. The best water generation rates were observed for F-ZnO/SiO_2_-2L film (1.5–2.2 mL·g^−1^·day^−1^). It contained green ZnO and green silica made of peanut–banana biomass. Thanks to its surface polarity, hierarchical porosity, and tight inorganic-polymer interaction, the film provided repeatable adsorption–desorption cycles without losing its properties [34]. The outcomes from this research illustrate that, apart from minimizing the amount of chemicals used in the process, green synthesis techniques improve the function of the material used in producing passive water, thereby leading to more efficient results [77].

### 3.6. Decision-Matrix Evaluation

For the purpose of facilitating a fair comparison among all the chitosan-based nanocomposites for their multifunctional properties, a multi-criteria decision matrix (DM) model (Section 2.8) was used. The experimentally recorded variables were normalized on a scale of 0 to 1, weighted based on their level of significance towards water harvest from air, and added up to calculate the overall performance measure (S_total_). The details of this have been shown in Table 3. The sustainability measure is a qualitative parameter related to the source being renewable, partial replacement of synthetic chemicals with plant extractives and the energy consumption of the synthesis process.

The F-ZnO/SiO_2_-2L film, which consisted of 10 wt.% G-ZnONPs and 10 wt.% G-SiO_2_NPs, showed the best overall performance (S_total_ = 1.00). Such outstanding performance is due to the combination of hydroxyl-functionalized Zn-OH and Si-OH surface groups, leading to low water contact angles (43 ± 1.1°), large working adsorption capacity (Δq = 0.70 ± 0.03 g·g^−1^), and maximum passive water output rate (2.0 mL·g^−1^·day^−1^) with negligible structural deterioration (<2%) during humid cycles. The F-ZnO-SiO_2_ system had the second-best performance (S_total_ = 0.92), demonstrating that green–green mixed films maintain excellent hydrophilicity, adsorption ability, and robustness despite relatively low nanoparticle concentrations. The F-FeZnSi/ZnO-2L composite had the third-best performance (S_total_ = 0.84), owing to its designed hydrophilic gradient structure, which facilitates vapor condensation on the surface and effective drainage. Generally, the analysis performed by the decision matrix proved that semi-green nanomaterials, especially those combining green silica with ZnO, offered the best combination of hydrophilicity, adsorption capacity, water production, stability, and sustainability. This study revealed that the synergy among oxides and structural engineering plays a vital role in developing efficient, sustainable materials for atmospheric water collection systems [78,79].

## 4. Conclusions

The present work encompasses a detailed comparative study of different kinds of nanomaterials that are green in nature. This includes zinc oxide, silicon dioxide isolated from the peanut-banana biomass, FeZnSi and chitosan nanoparticles, all incorporated into a chitosan-based multifunctional matrix designed for humidity-responsive films for water harvesting purposes. The utilization of semi-green methods using natural acids/alkali derived from Phoenix dactylifera and banana peel facilitated the synthesis of oxide and composite nanomaterials with desired morphology, hydroxyl functional groups, and high surface area (115–188 m^2^/g). The characterization findings showed that the green nanomaterials exhibited defect-rich porous structures, which significantly enhanced their interfacial compatibility with the chitosan matrix. The wettability analysis indicated an enormous enhancement in the surface hydrophilicity when compared to pristine chitosan films through the lowering of the water contact angle from 82° to a minimum of 43°. The humidity cycling tests confirmed the capability of the system to attain reversible water sorption/desorption cycles at a working capacity (Δq) of 0.37–0.70 g·g^−1^, highlighting the critical role of chitosan as a hydrophilic, film-forming biopolymer that enables structural integrity, moisture responsiveness, and long-term cycling stability. Among all samples tested, the bilayer film composed of Chitosan/G-ZnO (10 wt%) on the top layer and Chitosan/G-SiO_2_ (10 wt%) in the bottom layer exhibited superior properties. This film achieved the highest passive water yield (2 mL·g^−1^·day^−1^), minimal weight loss (<2%), and demonstrated robust thermal and structural stability. Decision-matrix analysis further supported its superiority (S_total_ = 1.00), closely followed by the combination of G-ZnONPs and G-SiO_2_NPs (S_total_ = 0.92). The outstanding performance of green silica-ZnO nanocomposites is attributed to the synergistic interaction between Si-OH and Zn-OH groups on their surfaces, which enhances polarity, facilitates efficient hydrogen bond formation, and establishes well-regulated hydrophilic gradients in bilayer films. Importantly, chitosan acts not only as a passive matrix but as an active functional component that governs nanoparticle dispersion, interfacial adhesion, and moisture transport pathways, thereby amplifying the overall AWH performance of the system. In conclusion, the results show that semi-green oxide-polymer nanocomposites can be used as effective substitutes for synthetic counterparts possessing similar characteristics. These nanocomposites provide either equivalent or better results regarding the adsorption of atmospheric water vapor and have distinct ecological benefits. Silica-ZnO green nanocomposites embedded in chitosan films are among the most prospective and economical alternatives in this regard.

As a result, this research has effectively set the stage for green-synthesized bilayer films as an efficient method for atmospheric water harvesting, but future research needs to be directed towards the feasibility and stability of biomass extraction processes and subsequent testing of these nanocomposites’ durability in changing environmental conditions. Moreover, incorporation of photothermal or conductive green nanomaterials in the polymer composite would allow enhanced water harvesting due to an active water desorption cycle. Detailed insight into the bonding process between green oxide nanoparticles and the chitosan film through computational modeling is necessary to achieve efficient and sustainable AWH systems.

## Figures and Tables

**Table 1:** Summary of the film systems, their compositions, nanoparticle loadings, and structural architectures.

Film Code	Composition/Nanomaterial System	NP Loading (wt%)	Architecture
F-CH	Neat chitosan	0	Single-layer
F-ZnO	Chitosan/G-ZnONPs	10	Single-layer
F-SiO_2_	Chitosan/G-SiO_2_NPs	10	Single-layer
F-FeZnSi-NC	Chitosan/FeZnSi-NC	10	Single-layer
F-ZnO-SiO_2_	Chitosan/(Mixed G-ZnO-G-SiO_2_)	5 + 5	Single-layer blend
F-FeZnSi/ZnO-2L	Chitosan/FeZnSi-NC (top) Chitosan/G-ZnO (bottom)	5 + 5	Bilayer
F-ZnO/SiO_2_-2L	Chitosan/G-ZnO (10 wt%) (top) Chitosan/G-SiO_2_ (10 wt%) (bottom)	20 (total)	Bilayer (high-load)

**Table 2:** Surface Free Energy calculations derived via the OWRK Model for selected chitosan nanocomposite films, detailing dispersive and polar contributions.

Film Code	$\gamma_{s}^{d}$ [mN/m]	$\gamma_{s}^{p}$ [mN/m]	$\gamma_{s}$ [mN/m]
F-CH	28.4	12.1	40.5
F-SiO_2_	26.2	34.5	60.7
F-FeZnSi-NC	25.1	41.3	66.4
F-ZnO/SiO_2_-2L	24.8	48.2	73.0

**Table 3:** Decision-matrix evaluation of chitosan-based nanocomposite films, including experimental properties, normalized scores, total score (S_total_), and ranking.

Film System	WCA (°)	Δq (g·g^−1^)	Water Yield (mL·g^−1^·day^−1^)	Mass Loss (%)	Normalized Scores	S_total_	Rank
F-ZnO	79 ± 1.3	0.62 ± 0.02	0.35	2–3	0.60/0.89/0.17/0.75/0.80	0.67	5
F-SiO_2_	56 ± 1.2	0.56 ± 0.02	0.65	2–3	0.80/0.80/0.32/0.80/1.00	0.79	4
F-FeZnSi/ZnO-2L	61 ± 1.2	0.66 ± 0.02	0.60	2–3	0.70/0.94/0.30/0.80/0.90	0.84	3
F-ZnO-SiO_2_	58 ± 1.6	0.64 ± 0.02	1.00	1–2	0.95/0.92/0.60/0.90/1.00	0.92	2
F-ZnO/SiO_2_-2L	43 ± 1.1	0.70 ± 0.03	2.00	1–2	0.93/1.00/1.00/0.95/1.00	1.00	1

## Data Availability

Unprocessed data were available upon request from the corresponding author.
